# Antidiabetic Food-Derived Peptides for Functional Feeding: Production, Functionality and In Vivo Evidences

**DOI:** 10.3390/foods9080983

**Published:** 2020-07-23

**Authors:** Fernando Rivero-Pino, F. Javier Espejo-Carpio, Emilia M. Guadix

**Affiliations:** Department of Chemical Engineering, University of Granada, 18071 Granada, Spain; fjespejo@ugr.es (F.J.E.-C.); eguadix@ugr.es (E.M.G.)

**Keywords:** bioactivity, enzymes, glycemic index, nutraceutical, peptides, proteases, protein hydrolysates

## Abstract

Bioactive peptides released from the enzymatic hydrolysis of food proteins are currently a trending topic in the scientific community. Their potential as antidiabetic agents, by regulating the glycemic index, and thus to be employed in food formulation, is one of the most important functions of these peptides. In this review, we aimed to summarize the whole process that must be considered when talking about including these molecules as a bioactive ingredient. In this regard, at first, the production, purification and identification of bioactive peptides is summed up. The detailed metabolic pathways described included carbohydrate hydrolases (glucosidase and amylase) and dipeptidyl-peptidase IV inhibition, due to their importance in the food-derived peptides research field. Then, their characterization, concerning bioavailability in vitro and in situ, stability and functionality in food matrices, and ultimately, the in vivo evidence (from invertebrate animals to humans), was described. The future applicability that these molecules have due to their biological potential as functional ingredients makes them an important field of research, which could help the world population avoid suffering from several diseases, such as diabetes.

## 1. Introduction

### 1.1. Proteins, Protein Hydrolysates and Peptides

Proteins are one of the main components of human diets. These biomacromolecules are formed by the association of amino acids, through the peptidic bond between the amino group of one amino acid and the carboxyl group from the following amino acid in the chain [1]. Food proteins are an important topic in the research, due to their health benefits in human and their enormous variety.

At the end of the 20th century, scientists started to focus on studying the hydrolysis of food proteins in order to determine their forming-peptides, because some of them have been proven to be bioactive, and to have beneficial consequences in the organism [2]. The gastrointestinal digestion of proteins leads to the formation of peptides in the human tract due to the action of digestive proteases, and these have beneficial effects. However, the key in this topic is that different food-grade proteases might have different specificities [3], and from the same substrate, the pool of peptides produced would be different, and would show different properties.

A protein hydrolysate is the mixture of peptides that originally formed the protein, after its hydrolysis. The complex structure of proteins in the native state hides the functionality of the peptides, preventing them from exerting their bioactivity by association with some other molecules.

The hydrolysis reaction consists of breaking the peptidic bonds and releasing different-sized peptide chains, whose properties may well vary depending on the properties of the amino acids included in each peptide [4]. The released peptides usually present improved technological and biological properties that allow the utilization of these products to improve food formula properties. Protein hydrolysis leads to an improvement in different technological aspects: solubility, emulsifying and foaming capacity, water holding capacity, oil binding capacity and lipid oxidation prevention. The technological property modification of protein hydrolysates, compared to the intact protein, is an advantage related to their use in functional feeding, because it mainly involves the facilitation of the formulation of the food. This improvement is due to the exposition of the residues of different amino acids. In this context, both the molecular weights of peptides and amino acid sequences are important.

The solubility of proteins depends on the interactions between the macromolecules forming the protein [5]. Protein hydrolysis leads to polar group exposition when small peptides are released, and consequently, a high degree of hydrolysis is correlated with the higher solubility of protein hydrolysates [6,7]. This increase in solubility is important in the production of food and beverages intended for parenteral or gastric administration.

Proteins are amphipathic molecules due to the different polarities of the amino acids that compose them [8], which are absorbed into the interface formed during the emulsification process [9], reducing the interfacial tension and stabilizing the emulsion. Notably, limited hydrolysis leads to this interfacial activity, increasing the emulsifying activity [10]. Foaming property improvement also depends on the surface activity of the proteins [6], and low degrees of hydrolysis are adequate for an increase in foaming capacity [11]. Protein hydrolysis also enhances the water holding capacity and the oil binding capacity, depending on the amino acids composing the peptides and the lengths of them. Hydrophilic groups would retain water more effectively [12], whereas for oil binding capacity, the hydrophobic residues of proteins are important due to the lipophilic character of oils, thus allowing the interaction with their hydrocarbon chains [13]. These are important parameters to consider when formulating a fortified food product, because they would affect its structure.

Proteins and peptides show antioxidant activity [14,15]. Numerous protein hydrolysates have been reported as antioxidants, coming from different protein sources. Concerning technological improvement, it is important to remark that these peptides with antioxidant activities and emulsifying properties can be used in complex food matrices as an emulsion, those peptides being a double-action agent [16,17]. As such, these peptides would prevent the lipid oxidation process in food formulation [18]. Another improvement provoked by hydrolysis is the increase in digestibility and the loss of antigenicity of the proteins. This is an important statement in terms of nutrition; for example, for specific groups of people, such as infants or elder. The digestibility of proteins is increased because the available N-terminal sites are increased after enzymatic hydrolysis [19,20], and consequently, the peptidases action is enhanced. The antigenicity of proteins is caused by epitopes, specific sequences in the allergen proteins that are potentially recognizable by the immune system and would potentially activate an allergenic response. Enzymatic hydrolysis leads to allergenic epitopes degradation, and therefore helps to reduce the immunoreactivity of the native protein [21]. Enzymatic hydrolysis, due to its reaction conditions, does not destroy amino acids, which is desirable for food formulation because the nutritional profile of the proteins is conserved.

Nonetheless, one disadvantage that enzymatic hydrolysis can produce in terms of nutrition is the bitter taste of peptides, related to the release of hydrophobic residues. To overcome this limitation, the encapsulation of peptides inside different matrices (i.e., chitosan, glucose syrup) or the addition of flavor-developing proteases (i.e., flavourzyme) are adequate solutions. These techniques would avoid the disadvantages of traditional techniques, such as the adsorption of bitter peptides onto activated carbon, chromatographic removal, or selective extraction with alcohols [22].

On the other hand, the similarity of food-derived peptides to the structure of human regulatory peptides also makes them suitable for interacting with some enzymes and receptors involved in human metabolism. In this way, the most important improvement of proteins after hydrolysis, concerning functional food, is the bioactivity development. Lately, the proportion of the world population suffering an illness has increased, and prevention and pre-treatment are considered good options for most of them. At the economical level, the cost savings, compared to those associated with the treatment of the disease, are high [23]. Bioactive peptides are considered to be regulator molecules operating at different levels in the organism. As was previously mentioned, protein hydrolysis during digestion releases peptides that exert bioactivity in humans, but the intake of peptides with improved bioactivity, compared to those obtained naturally, is seen as a good option for humans [19]. This is due to the specificity of food-grade proteases employed in the industry, which are able to release peptides that digestive proteases cannot.

The bioactivity of peptides is considered to be related to the hydrophobicity characteristics of the residues, and more precisely, to the amino acid functional groups of their sequences [20]. Focusing on the bioactivities those peptides can exert, their antioxidant, antihypertensive and antidiabetic (glycemic index regulation) activities are the most remarkable because of the diseases they would prevent, which are some of the most prevalent worldwide nowadays (cardiovascular disease, diabetes, hypertension). Recent studies, both in vitro and in vivo, show the functionality of these protein hydrolysates. In vitro analyses allow comparisons of the biological potentials of different products, by evaluating, for example, the inhibitory capacity of different enzymes involved in metabolic processes.

Bioactive peptides can exert physiological effects at a cardiovascular, digestive, endocrine, immune and/or nervous level [24]. The most studied historically are antihypertensive and antioxidant, whereas there is less information regarding the antidiabetic properties of peptides coming from food proteins. Peptides are considered to be bioactive in different metabolic pathways, depending on how they interact with the human body. When it comes to inhibiting an enzyme, peptides can interact at the active site and/or outside the catalytic site of it, preventing the enzyme from interacting with the substrate.

Peptides are defined as antihypertensive when they are able to inhibit the angiotensin-converting enzyme (ACE). This enzyme plays a key role in blood pressure regulation [25], and its inhibition has a positive effect on hypertensive patients [26]. In the antioxidant case, a wide variety of mechanisms and implications are involved. Beyond their ability to slow down lipid oxidation in food systems, these peptides would also prevent oxidative stress related to several diseases such as hypertension and ageing [27]. As such, peptides with both antihypertensive and antioxidant activities can be considered anti-ageing peptides. Beyond these two bioactivities, anticholesterolemic, antithrombotic and anti-inflammatory peptides have also been described [28,29]. Furthermore, peptides might have diverse bioactivities, and might consequently exert synergistic effects on the human body. For example, a correlation has been proposed between diabetic and hypertensive patients, so a treatment for both problems would be ideal. Ketnawa et al. [30] obtained both ACE- and dipeptidyl peptidase IV (DPP-IV)-inhibitory peptides from rainbow trout. In this review, we will focus on glycemic index-regulating peptides, that is, antidiabetic peptides.

### 1.2. Carbohydrates Digestion Process and Diabetes

**Figure 1:**
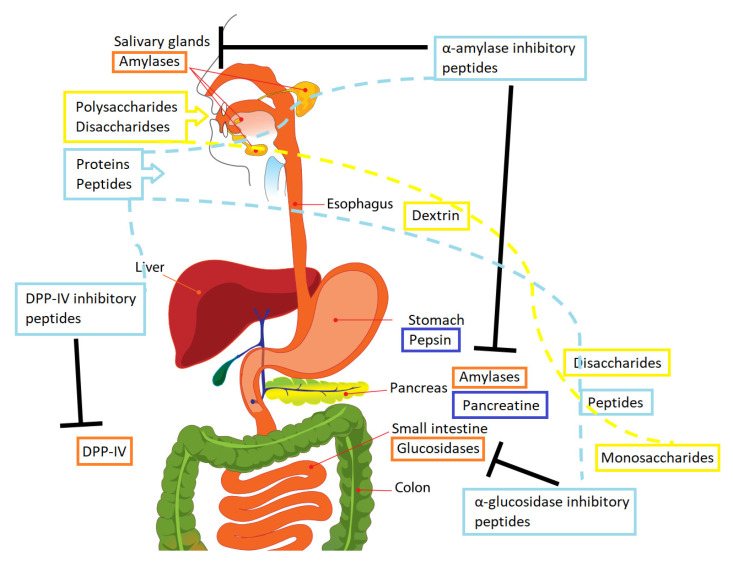
Mechanisms involved in peptides and carbohydrate digestion. The broken lines recreate the digestion process of the different molecules. Color reference: Yellow—Molecular state of carbohydrate during digestion; Orange—Main digestive enzymes involved in the carbohydrate digestion; Light blue—Molecular state of proteins during digestion; Dark blue—Digestive proteases. Permission: The original picture was released into the public domain by its author (LadyofHats) and modified by authors to depict the information detailed in the document.

The metabolism of carbohydrates is the process of transforming the carbohydrates ingested from food into glucose molecules, the most efficient source of energy. The carbohydrates in foods generally appear as polysaccharides, such as starch or cellulose, or as disaccharides, such as lactose or sucrose. Carbohydrate digestion involves different enzymes and a complex series of metabolic processes. A graphical simplification is depicted in Figure 1. Initially, when the bolus is ingested, digestive enzymes would hydrolyze these complex polysaccharides.

α-Amylase (EC 3.2.1.1) hydrolyzes complex carbohydrates such as starch into oligosaccharides, which would be further hydrolyzed by α-glucosidase. This enzyme is secreted from the salivary and pancreatic glands.

α-Glucosidase (EC 3.2.1.20) is a membrane-bound enzyme found in the epithelial mucosa of the small intestine (brush border of the enterocytes). It releases free glucose molecules from terminal, non-reducing (1-4)-linked α-glucose residues.

Furthermore, food intake causes the release of intestinal hormones called incretins (gastric inhibitory polypeptide, GIP, and glucagon-like peptide-1, GLP-1). These two would affect numerous target tissues in the body, acting as endocrine signals to the pancreas, leading to insulin production in the β-cells and the suppression of the release of glucagon in the α-cells. These two incretins are responsible for ~70% of the insulin secretion after meal intake [31]. This results in the uptake of glucose by the muscles, as well as a lower production of glucose in the liver. The final consequence is therefore the decrease in blood glucose after ingestion, which allows the adequate regulation of postprandial blood glucose levels. At this level, the enzyme dipeptidyl peptidase IV (DPP-IV) regulates the degradation of incretins according to physiological needs. GLP-1 and GIP have half-lives of approximately 2 min and 5–7 min respectively, before they are degraded by DPP-IV [32,33]. DPP-IV is a cell surface (EC 3.4.14.5) that cleaves dipeptides from the N-terminus of polypeptides, in which proline is at the penultimate position [34]. DPP-IV can largely be found on the luminal surface of enterocytes; therefore, it can interact with any of the molecules from food intake before their absorption, that can be further metabolized before the molecules’ interaction with soluble and vascular endothelial DPP-IV (the one affecting GIP and GLP-1 levels). Gut hormones released from the enteroendocrine cells play an important role in food intake regulation [35].

Diabetes mellitus type 2 is one of the most prevalent diseases, affecting more than 400 million people and with estimations of 700 million people becoming affected by 2045 [36]. This metabolic disorder is characterized by insulin resistance, that is, the inability of the organism to react to the insulin action, or an insufficient production of this hormone. It is especially important to control the postprandial glucose level, because the long-term consequences of high glucose levels in the bloodstream are diverse, from renal failure to neurological damage and cardiovascular disorders [24,37]. Regarding the causes, both genetic and environmental factors take part in diabetes development. It is believed that the main cause is obesity, which operates through several pathways including an imbalance in the concentration of hormones, cytokines and other inflammatory signals [33].

### 1.3. Diabetes Prevention Strategies

Numerous strategies to manage postprandial hyperglycemia, and consequently prevent the development of type 2 diabetes, have been described [24]. Insulin injection [38] is the direct treatment for this disease, positively regulating the functioning of the organism. The main disadvantage is that insulin cannot be orally ingested. In addition to this, medications involved in the metabolic pathway of digestion are also options as regards preventing and treating the disease. A graphical simplification of the most important mechanisms involved in diabetes prevention is depicted in Figure 1.

Food-derived peptides from food proteins play a crucial role in the regulation of glucose homeostasis, due to their implication at different levels (e.g., glucagon-like peptide 1 regulation) and due to their capacity to inhibit digestion-related enzymes. Furthermore, some authors have described peptides as being able to enhance cholecystokinin levels, a gut hormone regulating food intake [39,40]. Peptides and amino acids would have an effect on body fat loss, insulin secretion and glycaemia reduction, but further research is needed in order to unravel these mechanisms. Further information regarding the peripheral regulation of food intake can be found in the following references [35,41], including how protein digestion products act as signaling molecules in enteroendocrine cells.

Regarding the digestion process and the enzymes involved in carbohydrate metabolism, the first approach to preventing an increase in glucose blood level is to avoid the degradation of polysaccharides into glucose. Therefore, the inhibition of digestive hydrolases (amylases, glucosidases) would avoid complex polysaccharides from becoming hydrolyzed, and thus absorbed in the bloodstream. Amylases inhibition can be exerted in the saliva and in the gastrointestinal tract, lowering the blood glucose level [32]. For its part, glucosidase inhibition would essentially preclude the uptake of glucose into the blood circulation, effectively decreasing postprandial hyperglycemia [24,42,43]. Delayed carbohydrate absorption is considered an adequate contributing factor in stimulating GLP-1 secretion, which would ultimately lead to the incretin effect.

Among the α-glucosidase and α-amylase main inhibitors, we find acarbone, miglitol and voglibose. However, numerous side-effects, such as gastrointestinal disturbances, stomach pain and flatulence, have been described for these drugs, and consequently this have limited their use as inhibitors [24]. The obtaining of inhibitors for these enzymes with no side effects is consequently an interesting research topic.

On the other hand, if the body suffers from insulin resistance, considering that DPP-IV acts by degrading incretins [44], one of the oral antidiabetic drugs used today is the group of DPP-IV enzyme inhibitors called gliptins [45]. The discovery that the enzyme DPP-IV inactivates more than 95% of GLP-1 has put it in the spotlight as a type 2 diabetes mellitus (T2DM) management therapy [46]. When DPP-IV is inhibited, the inhibitory action it has on incretins is suppressed, and the half-life of these incretins is increased. Protein intake can also elevate plasmatic GLP-1 levels [47]. This causes insulin secretion to be stimulated, in addition to inhibiting glucagon release [34], and the blood glucose level is adequately regulated. The first gliptin approved by the Food and Drug Administration (FDA) was sitagliptin, in 2006, and since then, more synthetic DPP-IV inhibitors have been approved, in spite of the adverse effects they may have. Among these, we can find nasopharyngitis, nausea, hypersensitivity, headache, skin irritations and the risk of acute pancreatitis [48,49]. Furthermore, their long-term safety remains unclear.

Although the previous strategies described are the most important in terms of health and the research related to bioactive peptides, some other ways to prevent diabetes have been described. There are different kinds of molecules acting in different organs, which also have antidiabetic effects via different mechanisms, such as insulin sensitizers, insulin secretagogues, GLP-1 mimetics or glizofins [32,33,50]. There is a need for further research since some peptides are able to stimulate incretin secretion, this effect being related (or not) to the DPP-IV inhibition. Peptides are able to interact at many physiological levels in the human body [51].

Recent studies show the importance of diabetes pretreatment in minimizing the economic impact of the disease treatment [52,53], beyond the health consequences it has on the patient. Bioactive peptides appear to be a good alternative for employment in functional foods as health-promoting ingredients. In the literature, the discovery of peptides coming from food proteins able to inhibit amylases, glucosidases and DPP-IV has been reported. These kinds of enzyme-inhibitory peptides are still in the basic research stage, and none have been approved by the FDA [32]. Bioactive peptides can also regulate glucose homeostasis due to their ability to regulate gut hormones [35]. As such, bioactive peptides for preventing the development of diseases are an important field of research, the interest in which is increasing, and which could have positive effects on the human health and economic levels.

## 2. Production of Glycemic Index-Regulating Protein Hydrolysates

### 2.1. Enzymatic Hydrolysis Reaction

Obtaining bioactive peptides from food proteins is preferably carried out by enzymatic hydrolysis rather than chemically, or via microbial fermentation. Chemical hydrolysis requires high temperatures and an extremely acidic or basic environment in order to destabilize the bond, and consequently, some amino acids are modified or even destroyed, meaning a loss in the nutritional value of the peptides. Microbial fermentation, to produce peptides, is not a reproducible technique, since there are some uncontrollable factors (i.e., enzyme levels, metabolism of microbes, etc.). However, genetic recombinant strains could help palliate these limitations [54]. In this review, we will focus on obtaining bioactive peptides via enzymatic hydrolysis.

Enzymatic hydrolysis requires mild reaction conditions, and is specific and controllable. The reaction itself is simple, needing the substrate (protein) and the enzyme(s) (protease(s)). The reaction conditions (pH and temperature) are determined by the protease, and many factors, such as enzyme/substrate ratio or substrate concentration, must be taken into consideration too. The optimal conditions for obtaining highly bioactive hydrolysates are usually achieved via different kinds of experimental designs [55,56,57]. The enzymatic hydrolysis is generally carried out in a jacketed reactor, under stirring, in order to ensure the homogeneity and constant temperature of the reaction.

In terms of large-scale production, some authors have produced protein hydrolysates at a pilot or semi-pilot plant scale. In this context, different hydrolysates have been produced from such sources as fish discards [58] for their valorization, and trials have confirmed the results of production obtained at lab scale, or from boarfish [59], which show strong DPP-IV inhibitory activities.

Lately, scientists have been testing enzymatic hydrolysis carried out after or during the application of non-thermal techniques, such as high pressure, ultrasound or microwave. The global conclusions are that high hydrostatic pressure and ultrasound pre-treatment improve the efficacy of enzymatic hydrolysis and the consequent release of bioactive peptides [60,61,62,63]. Regarding the protein structure, the tertiary and quaternary structures are generally affected by high-pressure treatment, while the secondary structures tend to be maintained. Nonetheless, we must consider the possibility that pressure treatment may lead to the denaturation of proteins, but also to aggregation or precipitation [64]. With respect to the primary structure, the application of pressure does not affect the covalent bonds, and so the sequences of amino acids are not lost [65,66].

The main parameter for characterizing protein hydrolysates is the degree of hydrolysis. This is defined as the proportion of cleaved peptide bonds compared to the original protein. The higher the degree of hydrolysis, the smaller the peptides size would be in the product obtained after the hydrolysis reaction. It is generally reported that bioactive peptides have a length of 2 to 20 amino acids.

### 2.2. Proteases

The enzymes responsible for protein hydrolysis are called proteases (EC 3.4.X.X), and they can be classified via where they catalyze the hydrolysis of bonds. They can be considered (a) endopeptidases, if the cleavage site is inside the protein, or (b) exopeptidases, if the cleavage sites are located at or near the ends of chains. The active site of the protease determines its substrate specificity, that is, the position where the hydrolysis will take place [67]. Then, the choice of the protease employed is essential, since it will define the degree of hydrolysis and the profile of released peptides [68].

Endopeptidases can be classified depending on their catalytic mechanism and their tertiary structure, considering the amino acid or metal present in the active site, such as aspartate, cysteine, metallo or serine-proteases. Exopeptidases can be classified as aminopeptidases, carboxypeptidases or dipeptidases [3,69]. Some examples of proteases widely employed in the industry are Subtilisin, a non-specific endo-peptidase, exerting its proteolytic activity over hydrophobic amino acids [70], Trypsin, a specific endo-peptidase, exerting its proteolytic activity over arginine and lysine residues [71], and Flavourzyme, a complex mixture of endo- and exo-peptidases, exerting its proteolytic activity over lineal chains, releasing small peptides and free amino acids [72]. Depending on the type of bioactive peptide desired, certain enzymes have been tested and considered as adequate proteases for obtaining these molecules. For example, a combination of Alcalase and Flavourzyme has been reported as a good enzymatic treatment for obtaining DPP-IV inhibitory peptides [73,74]. For α-glucosidase inhibitory peptides, trypsin has been reported as an adequate protease [75], and so has Alcalase [76]. Further research should be carried out, since different proteins might lead to different bioactive protein hydrolysates.

### 2.3. Protein Source

The substrates usually employed for protein hydrolysis are of natural origin, usually with a high protein percentage. The most-studied protein substrates for obtaining antidiabetic peptides to date are milk [42,77,78] and soy proteins [34,79], due to their high biological value compared to other proteins. One such example is seen in Lacroix and Li-Chan [80], who described the formation of DPP-IV inhibitors from dairy proteins, using 11 enzymes and different substrates.

Another valuable protein source is marine species. The literature reporting fish peptides with antidiabetic activity has been recently stated [59,81,82,83]. In this context, the use of fishing discards as protein sources for value-added products is important [55,59,83,84]. The scientific community considers enzymatic hydrolysis as a helpful option for revaluing these low-quality products and increasing their potential, as they have no side effects on the patient’s health.

In addition, by-products of the food industry, such as whey or gluten, with adequate protein content are also possible options for generating value-added products. Recently, vegetable protein sources such as peas or lupine have also been used, given the lower ecological impact they have [85]. Similarly, insect or algae proteins are being used today in the food industry for the production of food products [86,87]. These sources of proteins are reported to be sustainable sources with great potential for use in the food industry. There is already literature concerning the production of DPP-IV inhibitory peptides from these kinds of sources, such as *Palmaria Palmata* and brewers’ spent grain [75,88].

The main differences among substrates are their protein structure complexities and their amino acid sequences. Table 1 shows the amino acid profiles of different substrates considered as novel protein sources, such as insects. It can be observed that in some cases, the difference in the amount of a certain amino acid (g/100 g of substrate) is remarkable, and consequently the bioactivity related to the peptides released after hydrolysis is expected to be considerable. This statement is based on the fact that the bioactivity of peptides is mainly related to their amino acid sequence characteristics, that is, their hydrophobicity and/or length. The enormous diversity of substrates that are currently being used to obtain peptides with antidiabetic capacity is summarized in Table 2. It can be seen that marine, vegetable, insect or dairy sources are used, among others. The choice of the protein source used for the production of peptides must consider, in addition to the resulting bioactivity of the peptides, the environmental and economic factors during its production, via a life cycle assessment.

## 3. Identification of Bioactive Peptides

### 3.1. Fractionation

The concentration and purification of bioactive fractions from hydrolysates is an important step in industrial implementation. Because protein hydrolysates are composed of a mixture of different peptides, some of them bioactive, some of them not, their profitable concentration by different technologies is crucial. Moreover, identification of the actual bioactive sequences in these fractions would enable us to verify their actual bioactivity, and their bioavailability, stability and functionality in the context of nutrition. Table 2 shows a summary of the recent antidiabetic bioactive peptide publications (from journals annexed to Scopus). This table includes which analyses were carried out (in vitro inhibition assays, identification of peptides, verification of bioactivity with synthetic peptides, bioinformatic analysis, cell-based assays and in vivo assays with animal models). These protein hydrolysates contain a pool of peptides obtained by the cleavage of different enzymes, and their respective abilities to inhibit DPP-IV or digestive enzymes will determine how bioactive they are. There are numerous studies reporting the production of DPP-IV inhibitory peptides [34,96,146,147,148]. Numerous authors have also identified α-glucosidase inhibitory peptides [75,149,150,151,152] and α-amylase inhibitory peptides [97,153,154].

Having obtained a bioactive protein hydrolysate, different technologies allow the separation of peptides based on different physicochemical properties (molecular weight, polarity or charge). The main technologies employed for fractionation are chromatography or membranes. The next step would therefore be identification by mass spectrometry, bioinformatics analysis to verify the functionality of the peptides, and ultimately assays with the chemically synthesized peptide, to establish the actual bioactivity of the peptides identified. The workflow is described below.

Chromatography is a laboratory technique for separating compounds. There are numerous types of chromatography, distinguished by their characteristics. In terms of peptide purification, size exclusion chromatography (SEC) and reverse-phase chromatography (RPC) are the most widely used. These two separate peptides depending on their size and their hydrophobic characteristics, respectively. Usually, the combination of both techniques is adequate to obtain fractions that can be injected into a mass spectrometer (MS) so as to identify the peptides contained therein. For example, Rivero-Pino et al. [84] reported a higher DPP-IV inhibition for sardine peptides ranging from 400 to 1400 Da after fractionation with a Superdex Peptide 10/300 GL column, using a fast protein liquid chromatography system.

Membrane technology allows the separation of a sample into retentate and permeate. In this case, the pore size of the membrane would make the peptides separate into different fractions, depending on their molecular weight. Different molecular weight cut-off (MWCO) membranes would separate the peptides depending on their size, enabling one to identify the most bioactive fractions [155], which are usually the smallest ones, and to discard larger peptides, which are generally non-bioactive. Lacroix and Li-Chan [80] ultrafiltered dairy protein hydrolysates using an Ultracel Amicon ultrafiltration unit model 8400, with membrane MWCOs of 10 kDa, 3 kDa and 1 kDa, and reported a higher DPP-IV inhibitory activity for < 3 kDa fractions. For α-amylase inhibitory peptides, Ngoh et al. [156] fractionated a Pinto bean hydrolysate using centrifugal ultrafiltration filters with MWCOs of 100, 50, 30, 10 and 3 kDa, reporting a higher bioactivity in the < 3 kDa fraction.

Considering a large-scale production of hydrolysates, purification by membranes would be an adequate means of obtaining different-sized fractions.

### 3.2. Peptide Sequence Identification

The identification of peptides is generally carried out by mass spectrometry (MS) analysis [157] of the most bioactive fractions after chromatographic purification or membrane separation. MS is an analytical technique that measures masses of atoms and molecules after their conversion to charged ions, with or without fragmentation, by an ionization process. This process allows one to identify unknown compounds, and to elucidate their structure and chemical properties. Characterization is done by their mass to charge ratios (m/z) and relative abundances [158]. In this case, controlled fragmentation allows the determination of amino acid sequences in order to identify peptides. As stated in Table 2, numerous studies have identified glycemic index-regulating peptides via their ability to inhibit different enzymes.

A protein hydrolysate is a mixture of peptides, some of them bioactive and some others not. The importance of the identification resides in the fact that the full characterization of the peptides involved in regulating the disease enables the manufacturer to claim the health-promoting property of the fortified product.

α-amylase inhibitory peptides are not as broadly studied as the α-glucosidase and DPP-IV inhibitors described. Some authors have suggested that peptides with branched chains (such as Lys, Phe, Tyr and Trp) and cationic residues are preferably bound to α-amylase [76,159]. Further, Siow and Gan [160] identified three α-amylase inhibitory peptides, ranging from 17 to 23 amino acids length, that might have low bioavailability due to their large molecular weight. Ngoh and Gan [156] reported the importance of Gly or Phe at the N-terminal and Phe or Leu at the C-terminal. However, the α-amylase inhibitory peptides’ features should be further researched, in order to establish similar statements as those concerning the DPP-IV inhibitory or α-glucosidase inhibitory peptides.

Concerning the α-glucosidase inhibitory peptides, Ibrahim et al. [43] summarized the structural properties of α-glucosidase inhibitory peptides. What is remarkable is the importance of amino acids containing a hydroxyl or basic side chain at the N-terminal (which could be expected from trypsin hydrolysis), and of proline within the chain and alanine or methionine at the C-terminal. Nonetheless, factors such as the length of the peptide, its hydrophobicity and its isoelectric point are not extremely important. Ser-Thr-Tyr-Val (STYV) has been reported as the most potent glucosidase inhibitory peptide [43].

Diverse features have been described for DPP-IV inhibitory bioactive peptides, such as the hydrophobic N-terminal [48] ideally tryptophan [161], and proline or alanine as the penultimate N-terminal residue [162], or a low molecular mass [73,83]. Ketnawa et al. [30] concluded that cationic peptides, obtained by electrodialysis with an ultrafiltration membrane, were the most bioactive fraction of the hydrolysate analyzed. Among the 222 peptides analyzed by Liu et al. [48], over 88.4% had a molecular weight lower than 1000 Da, and more than half had one lower than 500 Da. Ile-Pro-Ile (IPI) has been reported as the most potent DPP-IV inhibitory peptide (IC_50_ = 5 μM) [59].

The identification of bioactive peptides is a key point in this field of research. However, there are still limitations to this procedure due to the high number of molecules (free amino acids, small-/medium-size peptides, polypeptides, oligomers, undigested proteins, etc.) contained in a protein hydrolysate. Considering the presence of high molecular weight molecules, it is sometimes hard to identify low molecular weight peptides (<4 amino acids length), which are usually those responsible for the bioactivity [163]. In this regard, bioinformatics analyses play an important role in the identification of bioactive molecules.

### 3.3. Bioinformatics Analysis

Bioinformatics analyses should be taken into consideration given their potential use in identifying, characterizing and producing bioactive peptides [164]. The most remarkable analyses described below are in silico analysis, molecular docking and the Quantitative Structure–Activity Relationship.

The first approach to identifying bioactive peptides is the employing of informatics tools that use knowledge about proteins and proteases. Thus, having the sequences of the protein and knowing the selectivity of the enzyme, one can expect to obtain the resulting peptides after the cleavage (Table 2, rows where the enzymatic treatment column includes the term in silico). This method has advantages concerning its feasibility, but it also has disadvantages regarding the numerous protein structures that a substrate can have, and the fact that, depending on the reaction conditions, the proteases can act one way or another. One application for this analysis would be in identifying in which protein we could expect to obtain a peptide that it is known to have antidiabetic properties. One database largely cited in the literature is BIOPEP [165], a software that can, for example, detect bioactive peptides in a sequence, or simulate how proteases would act over a protein. There are more bioinformatics tools, such as ExPASy-PeptideCutter or Enzyme Predictor, that are capable of performing virtual hydrolysis, that is, in silico digestion [164].

The molecular docking technique predicts the preferred conformation of a molecule, when bound to another in order to form a stable complex. It is usually employed to see how an identified peptide can bind with the enzyme. Different crystal structures of DPP-IV, α-amylase and α-glucosidase can be found in the RCSB Protein Data Bank. It is a good approach to execute a screening of the different compounds, so as to choose the best candidates [48] and to discover where the peptide would interact with the enzyme. The software widely employed for molecular docking and virtual screening includes AutoDock Vina [166] and pepATTRACT [167].

Quantitative Structure Activity Relationship (QSAR) is an informatics tool that tries to predict the activity of a molecule based on its molecular features. This is based on the idea that structure and activity are related, and consequently, similar structures may well have similar activities [168]. The combination of different bioinformatics techniques is a good initial approach to confirming the bioactivity of identified peptides.

Fitzgerald et al. [169] recently published a manuscript on the application of in silico approaches for the generation of milk protein-derived bioactive peptides, including DPP-IV inhibitory peptides. On the same issue, Lacroix and Li-Chan [170] carried out an evaluation of the potential role of dietary proteins as precursors of DPP-IV inhibitors, via an in silico approach. Further, a structure–activity relationship was developed so as to theoretically predict the potential bioactivity of DPP-IV inhibitory peptides [171].

Ibrahim et al. [172] constructed a library of possible α-glucosidase inhibitory peptides based on the structural requirements of these kinds of biopeptides, which were subjected to in silico simulated gastrointestinal digestion and to molecular docking with glucosidase and amylase, in order to choose which peptides would be highly bioactive. Mora et al. [173] employed numerous in silico tools to characterize the bioactivity, resistance to digestion, permeability, allergenicity and toxicity of Iberian dry-cured ham peptides. These recent publications show the importance of in silico analysis in the discovery and/or characterization of bioactive peptides.

## 4. Bioavailability In Vitro

One important step in the research into bioactive peptides is to verify that the molecules would exert their activity in the human organism [174]. The biological functionality of a peptide depends, consequently, on its bioavailability. These molecules must be resistant to peptidases present in the gastrointestinal tract, the brush border and the serum. Therefore, they must escape hepatic metabolization, which impedes them from reaching the site of action [175]. Bioactive peptides orally ingested are supposed to be exposed to the action of at least 40 different enzymes before reaching systemic circulation [176]. Depending on where the peptide would act, the bioactivity’s resistance to the human metabolism should be maintained until they reach their target.

The first approach to evaluating the efficacy of the protein hydrolysate is to simulate gastrointestinal digestion in vitro, and analyze the remaining bioactivity. The integrity of these molecules can be modified during the digestion process, before they reach their active site. At this level, it is important to establish if, in the case of further hydrolysis, the resulting peptides after digestion are still bioactive. The digestion process of the protein involves the sequential attacking of different proteases, and pH conditions at different levels:

(a) Stomach—acid pH in presence of pepsin, which specifically cleaves aromatic and hydrophobic amino acids;

(b) Intestine—basic pH in the presence of a mixture of enzymes, pancreatin, which shows trypsin, chymotrypsin and elastase activity, that is, the cleavage of arginine and lysine, as well as aromatic and aliphatic amino acids [177].

Publications reporting the effects of simulated gastrointestinal digestion can be consulted in Table 2 (rows including simulated digestion in the enzymatic treatment). The effect of simulated digestion on bioactive peptides is different depending on the substrate and the enzymatic treatment employed [82,99,142].

It is stated that > 3-kDa peptides are more likely to be degraded by gastrointestinal proteases than < 3-kDa peptides, but this behavior depends also on the amino acid sequence of the proteins. The terminal residues are an important factor determining their resistance [178].

After digestion, the peptides obtained would be absorbed in the enterocytes, where brush border peptidases can be found. This process depends mainly on their size and hydrophobicity. Hence, intact peptide absorption can occur via different mechanisms, from the enterocyte into the portal circulation, described below [175,178].

PepT1-mediated transport: Small peptides (di- and tri- peptides) resistant to hydrolysis would enter via peptide transporters located on the basolateral membrane, regardless of their amino acid sequence [179].

Paracellular route: Water-soluble peptides would pass between cells through tight junctions (no energy needed) [180].

Transcytosis: Hydrophobic peptides would require precise energy to diffuse through the brush border membrane of mucosa cells via three different procedures. Large lipophilic peptides would enter into the lymphatic system due to their inability to reach the portal system [175].

Nonetheless, in vitro simulated gastrointestinal digestion assays do not offer precise results because the physiological conditions are not considered, such as cellular permeability or the intestinal and brush border enzymes. For that reason, research concerning bioavailability usually involves using cell cultures for in situ analysis. The human adenocarcinoma colon cancer cell monolayer (Caco-2) is the most widely accepted in vitro model of intestinal permeability, due to its similarities with intestinal endothelium cells (human intestinal enterocytes) [181,182]. When cultured under specific conditions, Caco-2 cells form a continuous monolayer with a structural arrangement that serves as a model of both paracellular and transcellular movement. Numerous intestinal enzymes involved in food digestion are expressed on the surface of Caco-2 cells, including DPP-IV [34,183]. Therefore, it is recognized as an adequate model for drug absorption, toxicity testing and oligopeptide transport [175,182]. For instance, Zhang et al. [184] reported that a percentage of the DPP-IV inhibitory peptide IADHFL was degraded while passing through a monolayer of Caco-2 cells.

It is considered that di- and tripeptides are able to reach the systemic circulation via the transport means aforementioned, but these inhibitory peptides might exert their physiological effects over DPP-IV in the proximal small intestine, not in the plasma [77].

After that, serum peptidases in the human blood could ultimately degrade the peptides before reaching their target organ. Lammi et al. [34] developed a fast, sensitive and cost-effective ex vivo DPP-IV assay for human serum by collecting venous blood from a healthy female volunteer and analyzing how peptides would inactivate the enzyme, compared to sitagliptin as a positive control. The authors characterized the bioactive properties of a soybean peptide and a lupine one, overcoming the use of more expensive and less ethical in vivo approaches.

Specific in situ cell-based assays and ex vivo tools narrow the gap between the in vitro assays and the in vivo studies. Beyond all these physiological factors, the food matrix containing bioactive peptides would also have an effect on the bioavailability of molecules, since digestion depends also on the enzyme’s susceptibility to hydrolyzed peptides, which depends on the physical availability of it and the possible interactions occurring during digestion. This topic will be discussed in the following section.

The stability of peptides can be improved by different techniques. Gianfranceschi et al. [185] reviewed the biochemical peculiarities that can enhance the nutraceutical functionality of peptides, that is, their ability to be actually bioactive at their active site. Some techniques for the chemical modification of amino acids would prevent them from being digested by proteases, and consequently, peptide structure would be maintained, and the peptides would be expected to exert their physiological activity. Beyond that, trapping peptides inside different matrices increases their bioavailability too. For example, chitosan is a polymer able to increase the paracellular permeability of peptide drugs across mucosal epithelia [186]. Research on peptide absorption lacks studies regarding the influence of food matrices. It is important to investigate the influence of coexisting food components on the absorption of food-derived peptides. Harnedy-Rothwell et al. [187] subjected DPP-IV inhibitory peptides to simulated digestion in different matrices (tomato soup and juice), and verified that bioactivity was conserved. Different food matrices influenced protein and peptide digestibility during gastrointestinal digestion and absorption, so this must be considered a major factor in characterizing the bioaccessibility and bioavailability of peptides [174,188].

Unravelling the mechanisms that explain how nutrients might have physiological effects on the human body would allow to design or optimize the production of molecules with adequate molecular features for enhancing the bioactive properties of the ingredients [41]. In this sense, protein hydrolysates, sometimes poorly characterized, might have different bioactivities with synergistic effects responsible for the antidiabetic effect that they exert on humans.

Related research to be remarked upon includes the observation of a reduction in the gastrointestinal hydrolysis of a peanut protein isolate in the presence of polysaccharides, which is suggested to be due to the non-specific interactions between the polysaccharides and the peptides [189], or the reporting that almond flour inside a chocolate mousse and a sponge cake reduces protein degradation by pepsin [190]. In this regard, the effects of sugar-containing matrices could lead to Maillard product formation, and this would have an effect on the digestibility of proteins, since some amino acids are destroyed [191,192].

## 5. Stability and Functionality in Food Matrices

The food processing operations currently employed in the industry include thermal treatments (sterilization, pasteurization), non-thermal treatments (high-pressure homogenization or processing, ultrasound), storage (freezing and frozen), drying (dehydration, spray drying, freeze-drying) and separation (membrane processes). Some of these processes may well affect food protein functionality, due to physical and chemical changes. Proteins and peptides are prone to interact between one another, and with other molecules. The processing of food products containing proteins and peptides could, in consequence, reduce, maintain or enhance their bioactivity [54]. The amino acid residues would interact with molecules in different ways, also depending on the location of the peptides in the food matrix, ultimately affecting their native and denatured polymeric state [193,194]. It has been reported that high-fiber food matrices are adequate to carry these bioactive peptides, because chemical interactions are not likely to occur. A fiber network would avoid the aforementioned bitter taste of hydrophobic peptides, improving the sensorial acceptability of functional foods, including peptides [188,195].

There are not too many studies on how food processing and/or storage modify peptide structure, and consequently their functionality and bioactive properties. Graves et al. [196] analyzed the bioactivity of a rice bran peptide described as anti-cancer, during its 6-month storage inside an orange juice. Contreras et al. [197] reported some antihypertensive peptides’ resistance to atomization, homogenization and pasteurization, plus their retained bioactivity after incorporation into liquid yoghurt. Similar results concerning antioxidant and antihypertensive peptides’ resistance to food processing techniques were reported by Rivero-Pino et al. [198].

As aforementioned, some authors have reported that non-thermal treatments, such as ultrasound or high pressures, enhance protein enzymatic hydrolysis. However, there is a lack of information regarding how these processing techniques would affect peptides employed as ingredients in food formulation. These techniques are nowadays seen as less aggressive in terms of nutritional loss of ingredients [199], and are potentially employed for commercial sterilization or emulsifying processes.

Non-thermal processing technologies have been described to produce hypoallergenic foods due to structural epitopes changes [200], same as enzymatic hydrolysis [201]. The food industry can take advantage of this knowledge to fabricate hypoallergenic products without heat treatments. However, food processing may also affect amino acid composition, by forming derivatives such as lysinoalanine, d-amino acids and biogenic amines, which are usually related to undesired physiological consequences in the human body if consumed [202]. Hydrophobic amino acids tend to be more stable [203,204].

The consequences of known high-pressure treatments mainly affect the protein structure, leading to denaturation, aggregation or precipitation [64]. The effect must be studied for each case, considering the fact that the residue characteristics of peptides would be crucial in determining the result. Ultrasound reduces the size and hydrodynamic volume of the proteins, leading to better physical-chemical and emulsifying properties [205]. Drying processes improve the stability of products, extending the shelf-life of products by reducing water activity [206].

Another important example of chemical reaction is the formation of Maillard compounds, products of the non-enzymatic glycosylation of proteins. Sugar is a widely employed ingredient in the food industry due to its sweet flavor. The combination of reduced sugar with proteins or peptides at a high temperature leads to the formation of these compounds [207], affecting oxidative stability [208] and improving the antihypertensive or antioxidant bioactivities of protein hydrolysates [198,209,210,211,212]. Nonetheless, to the best of our knowledge, there is no literature reporting the increased DPP-IV inhibitory activity of Maillard reaction products coming from protein hydrolysates.

There are some techniques to avoid or slow down the effect of the digestion process, and to increase the peptides’ stability when introduced into food matrices. The most widely employed technique is encapsulation [213] with polymers or hydrogels [100].

Many factors are also involved in the potential loss or gain of bioactivity via the modification of peptides’ structure, or the aggregation of them. The state of the protein determines its properties [214], but the primary structure is not affected by the denaturation caused by most physical processes, [201], whereas in a more complex aggregation can occur. It is expected that protein hydrolysates, as a mixture of defined peptides, would not suffer further modifications, since the linear sequences are affected by sequence decomposition processes, such as hydrolysis itself or fermentation. The heterogeneous chemical composition of a food, as well as its molecular structure, is related to different chemical reaction behaviors [215] and, in consequence, its functionality.

Once the product containing peptides is formulated and its bioactivity maintained, it should also be ensured that bioactivity is not lost during its life as a commercial product. Chemical reactions might occur during the storage, depending on the formulation of the product and the temperature of it. The Maillard reaction has been described to occur at high temperatures, but long periods might lead to the appearance of Maillard reaction products too. Guyomarc’h et al. [216] reported the occurrence of the Maillard reaction within refrigerator-stored milk powder at 4 °C, whereas Albalá-Hutado et al. [217] reported it in liquid infant’s milk at room temperature. Recently, Harnedy-Rothwell et al. [187] fortified different food products (tomato-based soup and juice products) that were subjected to thermal treatments (sterilization and pasteurization) and stored at refrigerated temperature for 30 days. No modification of bioactivity was reported, indicating this treatment’s potential use on foods that could contain the bioactive protein hydrolysates.

Furthermore, peptides and proteins might tend to aggregate or precipitate over time, due to some other interactions, such as the van de Waals interaction, hydrogen bonding or a hydrophobic interaction. Hence, when considering the use of a protein hydrolysate as a bioactive ingredient, its stability during the food formulation, and its stability during storage, should be established.

## 6. Bioactivity Analysis

### 6.1. Bioactivity Initial Approaches

Nowadays, considering the novelty of the research subject, literature concerning in vivo analysis with animals and humans is extremely highly needed, but unfortunately, also scarce. Evidently, this research point is the most important, and is the one that offers authentic evidence concerning the implementation of these bioactive peptides as nutraceutical ingredients. The formulation of foods with legal claims to being a glycemic index-regulator due to the presence of these bioactive peptides would be the final step. For this purpose, plenty of evidence and verification in humans is required. The literature currently available on protein hydrolysates and bioactive peptides focusses mainly on in vitro analysis. In this regard, for the antidiabetic analysis, different analyses can be carried out, concerning the different metabolic routes involved in the disease. The most reported bioactive peptides with antidiabetic properties are those with amylases, glucosidases and DPP-IV inhibitory properties.

Concerning cell assays, among the cell lines generally used (Table 2) for the evaluation of the functionality of antidiabetic peptides, we found:

- BRIN-BD11: insulin-secreting cells (pancreatic B cells) in response to glucose, to analyze the effect of the compounds on insulin secretion [218];
- GLUTag: enteroendocrine cells that allow the secretion of GLP-1 (intestinal hormone regulated by the DPP-IV enzyme) to be measured using the ELISA technique [219];
- 3T3-L1: adipocyte cells that allow the measurement of glucose absorption by fluorimetry [220];
- STC-1: intestinal secretin tumor cell line that expresses and secretes gut hormones in response to physiological stimuli [221].

Different studies have employed these cellular lines in exploring DPP-IV inhibitory peptides, as can be observed in Table 2. For example, Harney et al. [81] showed that a blue whiting hydrolysate mediated insulin and glucagon-like peptide-1 (GLP-1) release from BRIN-BD11 and GLUTag cells, respectively, and Li et al. [122] observed the inhibition by a spirulina hydrolysate of the DPP-IV activity expressed by Caco-2 cells.

Nonetheless, in vitro, in situ and ex vivo approaches are not enough for the scientific community to establish claims about the functionality of food peptides.

### 6.2. In Vivo Analysis

In vivo analysis should be carried out to verify effectiveness, and to establish the required dose that should be consumed for the protein hydrolysate to effectively exert its biological activity. In these analyses, different markers are evaluated that indicate the physiological influence that these hydrolysates have on the subject [145,222]. In these investigations, model organisms, such as cell cultures or experimental animals, are used, while clinical studies in humans are less frequent. The results published so far are promising, since they show that, indeed, these protein hydrolysates have beneficial properties for the organism.

#### 6.2.1. Invertebrates Models

The use of *Caenorhabdtis elegans* as the model organism [223] in examining the functionality of bioactive peptides is not extensively reported in literature. Wang et al. [224] and Zhou et al. [225] reported a delay in senescence and stress resistance, and lifespan extension, respectively, through the antioxidant activities of bioactive peptides from *Angelica sinensis* protein and mussels (*Mytilus edulis*). Focusing on the antidiabetic activity of peptides, Zhu et al. [226] proposed an integrated microfluidic device, that resembles the hyperglycemic condition in diabetics, using this nematode as a model, and thereby investigated the responses after exposition to continuously high glucose concentrations in a physiologically relevant manner. These first approaches suggest that this easy-to-work nematode could also be employed [227].

Another model organism employed in the research is Drosophila [228]. In the same way, scarce information is available in the bioactive peptides field. Chen et al. [229] reported the up-regulation of antioxidant-related genes, a prolonged lifespan and the reduction of the accumulation of peroxide products when feeding the animal with crimson snapper scale peptides. To the author’s knowledge, no studies have been published concerning the antidiabetic properties of food-derived peptides in Drosophila.

#### 6.2.2. Vertebrates Models

The easiest animal models to work with in vivo are rats and mice. In these assays, different biological parameters are measured. In the case of antihypertensive peptides, blood pressure and plasma ACE and renin concentrations are measured [230]. Animal models of type 2 diabetes usually reflect insulin resistance and/or beta cell failure. Furthermore, many of them are obese, reflecting the human condition, wherein obesity is closely linked to type 2 diabetes development [231]. This latter author summarizes numerous examples of these animal models as related to diabetes.

In regard to the literature dealing with this topic, Harnedy et al. [81] and Parthsarathy et al. [98] reported a protein hydrolysate from blue whiting and boarfish with in vitro and in vivo antidiabetic properties, using cell cultures and mice. Similar research was carried out by Jung et al. [232] with silk fibroin hydrolysate, and by Hsieh et al. [162] with milk proteins. Mochida et al. [233] reported that zein-derived peptides induced glycemic regulation via GLP-1 secretion, and DPP-IV inhibition in rats, whereas Ishikawa et al. [234] obtained similar results by employing rice-derived peptides. Valencia-Mejía et al. [109] studied the antihyperglycemic and hypoglycemic activity of naturally occurring peptides and protein hydrolysates from beans in male Wistar rats.

D’Souza et al. [235] introduced an α-amylase inhibitor peptide into *Lactococcus lactis*, a bacteria usually employed to produce a yogurt, and diabetic mice fed with it showed a reduction in blood glucose levels after 20 days. Along the same line, Wang et al. [236] improved glycemic control in diabetic rats vis administration of fish skin gelatin hydrolysates, with a DPP-IV inhibitory capacity and a GLP-1 stimulation capacity. Drotningsvik et al. [237] showed that fish protein hydrolysates could affect different metabolic parameters, such as postprandial glucose regulation and lipid metabolism in obese Zucker rats.

Swine have been used as a model due to their similarities to human species. We share cardiovascular anatomies and functions, metabolisms, lipoprotein profiles, tendencies to obesity, etc., making swine adequate for testing the functionality of molecules altering metabolism [238]. The use of swine as an animal model for diabetes is stated [239,240]. However, studies wherein peptides are included in their diets do not focus on the bioactive effects on these features, but on the palatability or feed efficiency, in order to improve their nutritional status and gut function [177].

#### 6.2.3. Humans

Finally, the authentic evidence that bioactive peptides are adequate for employment in the food industry as nutraceuticals must overcome the clinical analysis carried out in humans. Peptides have extensive applications in medicine nowadays. The Food and Drug Administration (FDA) has approved more than 60 peptide drugs for marketing, and thousands of preclinical studies are being carried out for numerous peptides [241]. Concerning the regulatory requirements of protein hydrolysates from food proteins, different countries have developed different protocols to approve them as health-promoting ingredients [242]. For instance, at the European level, the European Food Safety Authority approved some angiotensin-converting enzyme inhibitory peptide products as a functional food ingredient, but no glycemic index-regulator peptides have been approved so far.

Concerning the bioactive peptides from food protein hydrolysates, studies are mainly carried out by employing dairy or fish proteins hydrolysates, since these are the most studied ones. Focusing on dairy proteins, the large amount of proline residues in casein makes this protein exceptional for the production of DPP-IV inhibitory peptides [24]. There are numerous studies reporting the efficacy of casein protein hydrolysates in humans, as a pretreatment for diabetes [145,243,244,245,246,247], which involve the observing of different parameters related to an adequate regulation of glucose blood level in type 2 diabetes patients. Recently, Saleh et al. [248] studied the effect of casein protein hydrolysate (a twice-daily dose of 8.5 g) in patients with gestational diabetes, concluding a moderate reduction of plasma glucose levels, suggesting the potential functionality of protein hydrolysate in the prevention of diabetes. Along the same line, whey [249,250,251,252] and egg [253] protein hydrolysates have been proven to have a positive effect on postprandial blood glucose, both in T2DM subjects and in healthy subjects. Calbet and Holst [254] reported that milk protein hydrolysates elicited about 50% more gastric secretion than the native protein, plus higher GIP plasma levels during the first 20 min of the gastric emptying process.

On the other hand, fish proteins are also seen as an adequate protein source [255]. Along this line, Hovland et al. [256] showed the effectiveness of milk and different fish protein hydrolysates (2.5 g/day of proteins) in affecting glucose regulation and acting as markers of insulin sensitivity in overweight adults, in a randomized, double blind study. Fish species, such as cod [257] or boarfish, proteins [258] have also been employed in human studies concerning diabetes prevention.

However, Curran et al. [259] showed the need for further precise nutrition analysis, after showing the ability of a casein hydrolysate to improve glycemic function only in some of the individuals analyzed.

All the aforementioned evidence shows that the enzymatic hydrolysis of food proteins is an adequate methodology for obtaining a mixture of peptides that are potentially bioactive. Historically, casein protein is the most widely studied protein, and there are currently food products including it as an ingredient. However, this ingredient in food products is not stated as a bioactive compound, but as a nutritionally improved protein. For example, Arla Foods Ingredients offers a range of whey protein hydrolysates, described as being more quickly absorbed into the blood, and Abbott declare that collagen protein hydrolysate, in their Promod^®^ Liquid Protein, helps to improve pressure ulcer healing [260]. Concerning antidiabetic hydrolysates, Nutripeptin^TM^ by Copalis Sea Solutions^®^ is described as a glycemic index-reducing peptide extracted by enzymatic hydrolysis from fresh or fresh-frozen fillets of codfish.

Protein hydrolysates’ functionalities as ingredients are currently an important topic. The biggest drawback concerning the bioactive properties of protein hydrolysates would be that the in vivo results show differences among individuals. Metabotyping individuals is an important step when considering which subgroups of people could benefit from protein hydrolysates as a functional food [261].

## 7. Conclusions

The available literature on bioactive peptides highlights their relevance to nutrition. The potential of bioactive peptides as antidiabetic agents to be employed in food formulation is a relevant field of research. A protein hydrolysate is a source of peptides capable of modulating different physiological processes. The choice of the protein source is essential, considering not only the bioactivity of peptides but also the environmental, economic and social factors. Then, its potential use as an ingredient must include the evaluation of its stability during storage, and its sensory properties via technical studies. Furthermore, there is a need to validate the antidiabetic properties of food-derived peptides through well-designed clinical trials with cellular assays, in animals and humans, to ensure their effectiveness and safety. These studies would describe the actual activity of protein hydrolysates with the purpose of being commercially developed in the food industry for functional feeding.

## Figures and Tables

**Table 1:** Amino acid content of some vegetable, insect and fish proteins (g/100 g of substrate).

Amino Acid	Quinoa	Lentil Protein Isolate	Brewer Spent Grain	Mealworm Larvae Meal	Silkworm Pupae Meal	Mussel Meal	Herring
**Essential**							
H	2.2	2.0	3.6	2.9	2.6	1.9	2.1
I	0.8	3.1	4.2	4.7	5.1	4.5	3.3
L	2.5	6.9	7.2	8.0	7.5	7.2	7.9
K	2.3	5.6	3.1	6.3	7	8.3	10.1
M	0.3	0.6	1.4	1.4	3.5	2.6	3.3
F + Y	2.8	7.1	9.7	9.5	11.1	8.7	5.9
T	5.7	3.0	3.2	4.3	5.1	5.3	4.0
W	1.0		-	1.2	0.9	1.0	-
V	1.0	3.5	6.0	8.5	5.5	4.6	4.6
C	0.1	0.5	1.4	0.8	1	1.0	1.1
**Non-essential**							
R	3.0	7.4	5.9	5.4	5.6	7.6	7.5
G	3.0	3.1	3.8	5.5	4.8	6.6	7.6
E	8.7	15.5	24.8	10.6	13.9	14.0	17.1
D	3.7	10.5	6.6	7.8	10.4	11.3	9.3
P	1.8	2.9	9.7	6.0	5.2	4.2	4.7
S	1.7	5.2	4.1	4.6	5.0	5.4	4.3
A	2.2	3.4	4.3	8.4	5.8	5.1	7.1
Ref.	[89]	[90]	[91]	[92]	[93]	[94]	[95]

A = alanine, R = arginine, D = aspartic acid, C = cysteine, E = glutamic acid, G = glycine, H = histidine, I = isoleucine, L = leucine, K = lysine, M = methionine, F = phenylalanine, P = proline, S = serine, T = threonine, W = tryptophan, Y = tyrosine, V = valine.

**Table 2:** Summary of recent publications concerning antidiabetic bioactive peptides (from Scopus, 2018–2020).

					In Vitro			Cellular Assay	In Vivo	
Substrate	Enzymatic Treatment	ID	Verification	B-A	DPP-IV	GIA	AMY	Cell line	Model	Ref
Rainbow trout (*Oncorhynchus mykiss*)	Alcalase	No	No	No	Yes	No	No	No	No	[30]
Camel whey protein	PTN 6.0S	Yes	Yes	Yes	Yes	No	No	No	No	[57]
Boarfish (*Capros aper*)	Alcalase 2.4 L, Flavourzyme 500 L; Simulated digestion	Yes	Yes	No	Yes	No	No	Caco-2; BRIN-BD11	No	[59]
Blue whiting (*Micromesistius poutassou*)	Alcalase 2.4 L and Flavourzyme 500 L Simulated digestion	Yes	No	No	Yes	No	No	BRIN-BD11, GLUTag, 3T3-L1	NIH Swiss mice	[81]
Cricket (*G. sigillatus*)	Alcalase Simulated digestion	No	No	No	Yes	No	No	No	No	[96]
Soybean (*Glycine max*)	Simulated digestion	Yes	No	No	Yes	Yes	Yes	No	No	[97]
*Luffa cylindrical* seed	Alcalase, trypsin	No	No	No	No	Yes	Yes	No	No	[76]
Salmon *(Salmo salar*)	Alcalase 2.4 L, Alcalase 2.4 L and Flavourzyme 500 L, and Promod 144 MG	Yes	No	No	Yes	No	No	BRIN-BD11; GLUTag	No	[73]
Boarfish *(Capros aper*)	Alcalase 2.4 L, Flavourzyme 500 L Simulated digestion	No	No	No	Yes	No	No	Caco-2; BRIN-BD11; GLUTag; 3T3-L1	Mice	[98]
Mealworm *(Tenebrio molitor*)	Alcalase, trypsin, ficin, flavourzyme	No	No	No	Yes	No	No	No	No	[74]
Tropical banded crickets *(Gryllodes sigillatus)*	Protamex Simulated digestion	No	No	No	Yes	No	No	No	No	[99]
Hempseed (*Cannabis sativa*)	Pepsin, trypsin	No	No	No	Yes	No	No	Yes	Ex-vivo	[100]
Bovine whey	Trypsin	Yes	Yes	No	Yes	No	No	No	No	[101]
Sea cucumber (*Stichopus japonicus*)	Simulated gastrointestinal digestion	Yes	No	Yes	Yes	No	No	3T3-L1, HepG2	No	[102]
Casein	PROTIN SD-NY10	No	No	No	No	Yes	Yes	No	No	[103]
Walnut (*Juglans mandshurica*)	Alcalase 2.4 L	Yes	Yes	Yes	No	Yes	Yes	HepG2 cells	No	[104]
Mealworm (*T. molitor*), locust (*Schistocerca gregaria*), cricket (*G. sigilatus*)	Simulated digestion	Yes	Yes	No	No	Yes	No	No	No	[105]
Corn germ	Alcalase, flavourzyme, trypsin	No	No	No	Yes	Yes	Yes	No	No	[106]
Millet grains (*Panicum miliaceum*)	Simulated digestion	Yes	No	No	No	Yes	Yes	No	No	[107]
Cowpea (*Vigna unguiculata)*	Alcalase + Flavourzyme	No	No	No	Yes	Yes	Yes	No	No	[108]
Beans (*Phaseolus vulgaris*)	Simulated digestion	No	No	No	No	Yes	Yes	No	Male wistar rats	[109]
Basil seeds (*Ocimum tenuriflorum*)	Pepsin	No	No	No	No	No	Yes	No	No	[110]
Brewers’ spent grain	Alcalase + Flavourzme Simulated digestion	Yes	Yes	No	Yes	No	No	No	No	[111]
*Porphyra dioica* extracted protein	Alcalase + Flavourzyme	Yes	Yes	No	Yes	No	No	No	No	[112]
Red Seaweed (*Porphyra spp*)	Alcalase, neutrase, pepsin, and trypsin	Yes	Yes	No	No	No	Yes	No	No	[113]
Soybean (*Glycine max*)	Trypsin	Yes	Yes	Yes	No	Yes	No	No	Mice	[114]
Rice albumin (*Oryza sativa japonica*)	Trypsin	No	No	No	No	No	No	STC-1	Wistar rats	[115]
Tuber storage proteins	Simulated digestion (*In silico*)	Yes	No	Yes	No	No	No	No	No	[116]
Rambutan (*Nephelium lappaceum*), pulasan (*N. mutabile*)	Simulated digestion	Yes	No	Yes	No	No	Yes	No	No	[117]
Pinto beans *(P. vulgaris*)	Protamex	Yes	Yes	Yes	No	No	Yes	No	No	[118]
Egg white ovoalbumin	Simulated digestion (In silico)	Yes	Yes	Yes	Yes	No	No	No	No	[119]
Salmon skin collagen (*Salmo salar*)	Pepsin, trypsin, papain, Alcalase 2.4 L	Yes	Yes	Yes	Yes	No	No	No	No	[120]
Quinoa *(Chenopodium quinoa*)	Papain, ficin, bromelain (In silico)	Yes	Yes	Yes	Yes	No	No	No	No	[121]
Spirulina (*Arthrospira platensis*)	Tryspin	Yes	No	No	Yes	No	No	Caco-2	No	[122]
Tomato seed proteins (*Solanum lycopersicum*)	15 enzymes (In silico)	Yes	No	No	No	No	No	No	No	[123]
Egg	Pepsin, trypsin (in silico)	Yes	Yes	Yes	Yes	No	No	No	No	[124]
Common carp (*Cyprinus carpio*)	Papain, neutrase, trypsin, pepsin; Simulated digestion	Yes	Yes	Yes	Yes	No	No	Caco-2 HepG2	No	[125]
Spirulina (*Spirulina platensis*)	Trypsin, pepsin	Yes	No	No	Yes	No	No	Caco-2	No	[126]
Pea (*Pisum sativum)*	Alcalase, neutrase	Yes	No	No	No	No	No	No	Male Kunming mice	[127]
Buffalo colostrum (*Bubalus bubalis*)	Simulated digestion	Yes	Yes	Yes	Yes	No	No	No	No	[128]
Chicken feet (*Gallus gallus domesticus*)	Neutrase, Protamex	No	No	No	Yes	No	No	STC-1	Wistar Rats	[129]
Portuguese Oyster (*Crassostrea angulata*)	Pepsin, bromelain, papain	Yes	No	Yes	Yes	No	No	No	No	[130]
Casein	Alcalase, protamex, neutrase, bromelain, and papain	Yes	Yes	Yes	Yes	No	No	No	Male Kun Ming mice	[131]
Whey	Corolase 2TS, Protamex	No	No	No	Yes	No	No	No	No	[132]
Soy (*Glycine max*)	Alkaline proteinase, papain, trypsin; Simulated digestion	Yes	Yes	No	Yes	Yes	No	No	No	[133]
Egg	Simulated digestion	Yes	Yes	Yes	Yes	No	No	Caco-2	Wistar rats	[134]
Rapeseed (*Brassica napus*)	Alcalase, trypsin pepsin, flavourzyme, papain	Yes	Yes	Yes	Yes	No	No	No	No	[135]
Lesser mealworm (*A. diaperinus*)	Simulated digestion; alcalase, Flavourzyme, papain, and thermolysin	Yes	No	No	Yes	No	No	No	No	[136]
Camel skin gelatin (*Camelius dromedarius*)	Alcalase, protease from S. *Griseus*	No	No	No	Yes	No	Yes	No	No	[137]
Chicken (*Gallus gallus*)	Corolase, Flavourzyme	Yes	No	No	Yes	No	No	Skeletal muscle	No	[138]
Kiwicha (*Amaranthus caudatus*)	Simulated digestion	Yes	No	No	Yes	No	Yes	Caco-2	No	[139]
Silver carp (*Hypophthalmichthys molitrix*)	Alcalase 2.4 L, neutrase, pepsin, trypsin, Flavourzyme	Yes	Yes	Yes	Yes	No	No	No	No	[140]
Flaxseed (*Linum usitatissimum*), rapeseed (*Brassica napus*), sunflower (*Helianthus annuus*), sesame (*Sesamum indicum*), soybean (*Glycine max*)	Subtilisin, pepsin, pepsin (In silico)	Yes	No	Yes	No	No	No	No	No	[141]
Bambara bean (*Vigna subterranean*)	Alcalase, thermolysin, trypsin Simulated digestion	Yes	No	Yes	Yes	No	No	No	No	[142]
Mealworm (*T. molitor*)	Pepsin, papain (In silico and experimental)	Yes	No	No	Yes	No	No	No	No	[143]
Yellow field pea (*Pisum sativum*)	Alcalase, chymotrypsin, pepsin, trypsin	Yes	No	No	No	Yes	Yes	No	No	[144]
Sardine (*Sardine pilchardus*)	Alcalase, Trypsin, Flavourzyme	Yes	No	Yes	Yes	No	No	No	No	[84]
Sodium caseinate	Simulated digestion	Yes	Yes	No	No	No	No	BRIN-BD11, 3T3-L1	Mice	[145]

ID: The reference includes the identification of bioactive peptides. Verification: The identified peptides’ bioactivities were confirmed with synthetic peptides. B-A: Any kind of bioinformatic analysis was carried out after identification of peptides. In vitro columns refer to inhibition assays of the following enzymes—DPP-IV: Dipeptidil-peptidase IV; GIA: glucosidase; AMY: amylase. Cellular assay: Cell-based analyses were carried out, referring to the cell line employed. In vivo refers to animal models studies. Note: Numerous references cited contain more analysis; only the antidiabetic properties analyzed were mentioned.
